# Crystal structure of dioxidobis(pentane-2,4-dionato-κ^2^ *O*,*O*′)[1-phenyl-3-(pyridin-4-yl)propane-κ*N*]uranium(VI)

**DOI:** 10.1107/S2056989014026607

**Published:** 2015-01-01

**Authors:** Takeshi Kawasaki, Takafumi Kitazawa

**Affiliations:** a Department of Chemistry, Faculty of Science, Toho University, 2-2-1 Miyama, Funabashi, Chiba 274-8510, Japan; b Research Center for Materials with Integrated Properties, Toho University, Miyama, Funabashi, Chiba 274-8510, Japan

**Keywords:** crystal structure, pentane-2,4-dionate, 1-phenyl-3-(pyridin-4-yl)propane, uranium(VI) complex

## Abstract

In the title compound, [UO_2_(C_5_H_7_O_2_)_2_(C_14_H_15_N)], the uran­yl(VI) unit ([O=U=O]^2+^) is coordinated to two acetyl­acetonate (acac) anions and one 1-phenyl-3-(pyridin-4-yl)propane (ppp) mol­ecule. The geometry around the U atom is UNO_6_ penta­gonal–bipyramidal; two uran­yl(VI) O atoms are located at the axial positions, whereas four O atoms from two chelating bidentate acac ligands and one N atom of a ppp ligand form the equatorial plane.

## Abstract 2

[UO_2_(acac)_2_(ppp)] is constructed from one uran­yl(VI) unit, two anionic acetyl­acetonate (acac) ligands and one 1-phenyl-3-(pyridin-4-yl)propane (ppp) ligand. The U atom exhibits a UNO_6_ penta­gonal–bipyramidal coordination geometry; two uran­yl(VI) O atoms are located at the axial positions, whereas four O atoms from two chelating bidentate acac ligands and one N atom of a ppp ligand form the equatorial plane.

## Chemical context   

The structural properties of uran­yl(VI) complexes are inter­esting from the viewpoint of nuclear fuels reprocessing and actinide waste treatment. In most commercial reprocessing plants, spent nuclear fuels are treated by the Purex method, in which uranium and plutonium are extracted from a nitric acid solution of spent nuclear fuels using tributyl-phosphate/*n*-dodecane. Uranium in the nitric acid solution exists as uran­yl(VI) ([O=U=O]^2+^) complexes. However, the Purex method has a few problems; for example, as the processing takes place on a relatively large scale, a large amount of extractant is necessary (Ikeda *et al.*, 2004 ▸; Suzuki *et al.*, 2012 ▸) Attempts to find other suitable coordinating ligands are therefore being undertaken. A number of structural studies of uran­yl(VI) β-diketonate complexes have been reported by ourselves and others (Alcock *et al.*, 1984 ▸, 1987 ▸; Huuskonen *et al.*, 2007 ▸; Kannan *et al.*, 2001 ▸; Kawasaki & Kitazawa, 2008 ▸; Kawasaki *et al.*, 2010 ▸; Sidorenko *et al.*, 2009 ▸; Tahir *et al.*, 2006 ▸; Takao & Ikeda, 2008 ▸). In particular, acetyl­acetonate (acac), is the simplest β-diketonate ligand and an important coordin­ating ligand for uranium. 
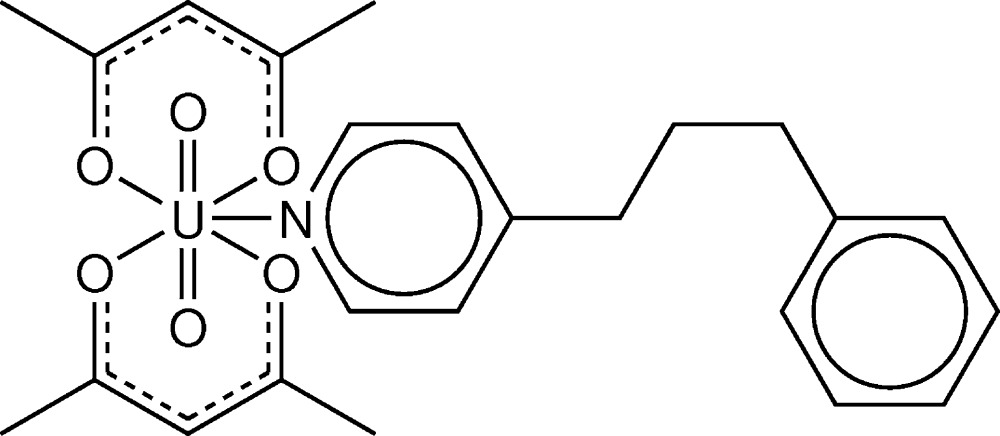


We report herein the synthesis and crystal structure of a novel uran­yl(VI) acetyl­acetonate (acac) complex with the pyridine-based ligand ppp [ppp = 1-phenyl-3-(pyridin-4-yl)propane] (Seth, 2014 ▸), namely, [UO_2_(acac)_2_(ppp)].

## Structural commentary   

**Figure 1:**
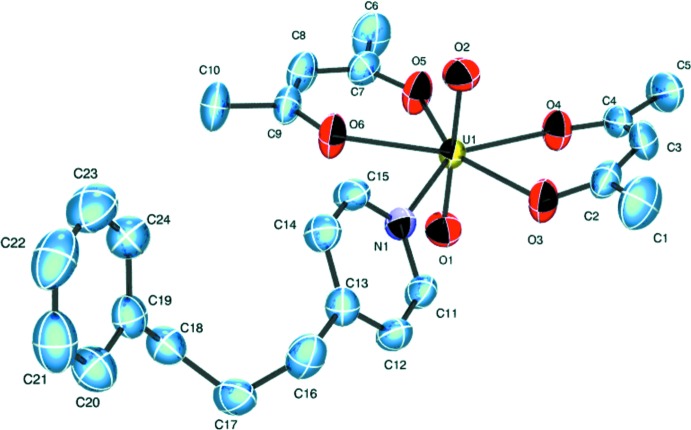
The mol­ecular structure of [UO_2_(acac)_2_(ppp)]. Displacement ellipsoids are drawn at the 50% probability level and H atoms have been omitted for clarity.

**Figure 2:**
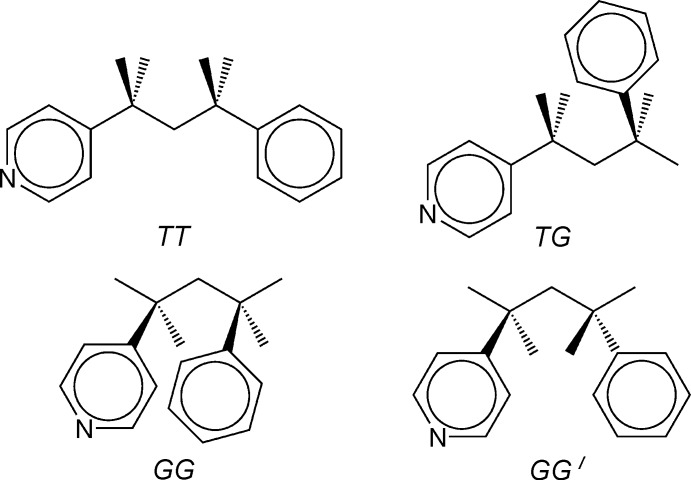
The four possible conformations that the ppp ligand can form (based on Carlucci *et al.*, 2002 ▸). In the title compound, the conformation is *GG*′.

The title compound of formula [UO_2_(C_5_H_7_O_2_)_2_(C_14_H_15_N)], is constructed from one uran­yl(VI) ([O=U=O]^2+^) unit, two acetyl­acetonate anions and one mol­ecule of ppp (Fig. 1 ▸). The uranium(VI) atom exhibits a penta­gonal–bipyramidal coord­ination geometry: two uran­yl(VI) oxygen atoms (O1 and O2) are located in the axial positions and four oxygen atoms (O3, O4, O5 and O6) from two chelating bidentate acac ions, together with one nitro­gen atom (N1) of the ppp mol­ecule, form the equatorial plane. The bond lengths around U1 (Table 1 ▸) decrease in the order U—N > U—O_acac_ > U=O. The dihedral angle between the pyridine ring of the ppp mol­ecule and the equatorial plane around U1 is 49.43 (12)°. The above structural properties are similar to those in the majority of previously characterised [UO_2_(acac)_2_ *L*] (*L* = pyridine derivative ligand) complexes (Alcock *et al.*, 1984 ▸; Kawasaki & Kitazawa, 2008 ▸; Kawasaki *et al.*, 2010 ▸). The conformation of the ppp mol­ecule is *GG*′ (Fig. 2 ▸). The dihedral angle between the pyridine ring and the phenyl ring in the ppp mol­ecule is 26.96 (13)°.

## Supra­molecular features   

**Figure 3:**
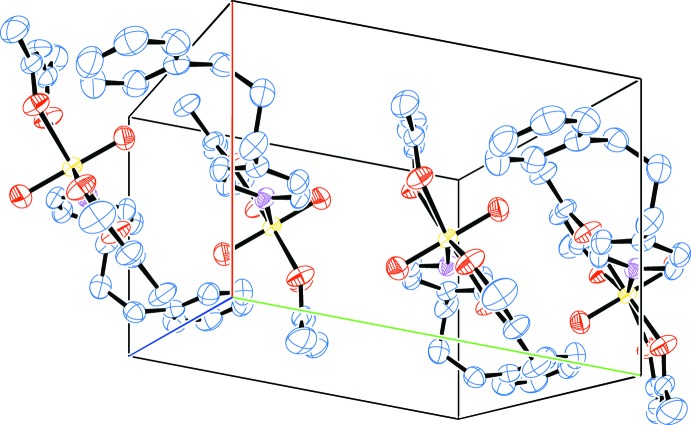
A packing diagram of the title complex (red line: *a* axis; green line: *b* axis; blue line: *c* axis). Displacement ellipsoids are drawn at the 50% probability level and H atoms have been omitted for clarity.

A packing diagram of title complex is shown in Fig. 3 ▸. The mol­ecules are stacked along the *b* axis, held together by van der Waals’ inter­actions only. Significant inter­molecular π–π and C—H⋯π inter­actions are not found.

## Synthesis and crystallization   

The title complex was synthesized according to literature procedures (Alcock *et al.*, 1984 ▸, 1987 ▸; Kawasaki & Kitazawa, 2008 ▸; Kawasaki *et al.*, 2010 ▸). To 10 ml of a methano­lic solution containing 1 mmol UO_2_(NO_3_)_2_·6H_2_O was added 3 mmol of acetyl­acetone and 3 mmol of 1-phenyl-3-(pyridin-4-yl)propane in 5 ml MeOH. The solvent evaporated slowly at room temperature for a few days and orange crystal were obtained.

## Refinement   

Crystal data, data collection and structure refinement details are summarized in Table 2 ▸. All H atoms were placed at calculated positions [C(CH)—H = 0.93, C(CH_2_)—H = 0.97 and C(CH_3_)—H = 0.96Å] and allowed to ride on their parent atoms with *U*_iso_(H) = 1.2*U*_eq_(CH,CH_2_) and *U*_iso_(H) = 1.5*U*_eq_(CH_3_).

## Supplementary Material

Crystal structure: contains datablock(s) I, global. DOI: 10.1107/S2056989014026607/cq2012sup1.cif

Structure factors: contains datablock(s) I. DOI: 10.1107/S2056989014026607/cq2012Isup2.hkl

CCDC reference: 1037284

Additional supporting information:  crystallographic information; 3D view; checkCIF report

## Figures and Tables

**Table 1:** Selected geometric parameters (, )

U1O1	1.773(3)	U1O5	2.348(2)
U1O2	1.777(3)	U1O6	2.354(2)
U1O3	2.330(2)	U1N1	2.610(3)
U1O4	2.360(2)		
			
O1U1O2	179.19(11)	O1U1N1	86.45(11)
O3U1O4	70.88(9)	O2U1N1	92.74(11)
O3U1O6	138.83(9)	O3U1N1	69.37(9)
O4U1O5	79.13(9)	O6U1N1	70.15(9)
O5U1O6	70.91(9)		

**Table 2:** Experimental details

Crystal data	
Chemical formula	[UO_2_(C_5_H_7_O_2_)_2_(C_14_H_15_N)]
*M*_r_	665.51
Crystal system, space group	Triclinic, *P* 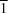
Temperature (K)	297
*a*, *b*, *c* ()	8.2100(16), 11.530(2), 14.516(3)
, , ()	108.67(3), 98.50(3), 100.81(3)
*V* (^3^)	1246.4(4)
*Z*	2
Radiation type	Mo *K*
(mm^1^)	6.55
Crystal size (mm)	0.47 0.29 0.26

Data collection	
Diffractometer	Bruker SMART APEXII
Absorption correction	Analytical (*XPREP*; Bruker, 2007 ▸)
*T*_min_, *T*_max_	0.149, 0.281
No. of measured, independent and observed [*I* > 2(*I*)] reflections	9353, 6948, 6026
*R*_int_	0.015
(sin /)_max_ (^1^)	0.722

Refinement	
*R*[*F* ^2^ > 2(*F* ^2^)], *wR*(*F* ^2^), *S*	0.027, 0.056, 0.99
No. of reflections	6948
No. of parameters	293
H-atom treatment	H-atom parameters constrained
_max_, _min_ (e ^3^)	0.88, 0.64

Computer programs: *APEX2*, *SAINT* and *XSCANS* (Bruker, 2007 ▸), *SHELXS97*, *SHELXL97* and *SHELXTL* (Sheldrick, 2008 ▸), *ORTEP-3 for Windows* (Farrugia, 2012 ▸) and *PLATON* (Spek, 2009 ▸).

## supplementary crystallographic information

### Crystal data

**Table:**

[U(C_5_H_7_O_2_)_2_O_2_(C_14_H_15_N)]	*Z* = 2
*M**_r_* = 665.51	*F*(000) = 640
Triclinic, *P*1	*D*_x_ = 1.773 Mg m^−^^3^
Hall symbol: -P 1	Mo *K*α radiation, λ = 0.71073 Å
*a* = 8.2100 (16) Å	Cell parameters from 4311 reflections
*b* = 11.530 (2) Å	θ = 2.6–28.5°
*c* = 14.516 (3) Å	µ = 6.55 mm^−^^1^
α = 108.67 (3)°	*T* = 297 K
β = 98.50 (3)°	Block, orange
γ = 100.81 (3)°	0.47 × 0.29 × 0.26 mm
*V* = 1246.4 (4) Å^3^	

### Data collection

**Table:**

Bruker SMART APEXII diffractometer	6948 independent reflections
Radiation source: fine-focus sealed tube	6026 reflections with *I* > 2σ(*I*)
Graphite monochromator	*R*_int_ = 0.015
Detector resolution: 8.333 pixels mm^-1^	θ_max_ = 30.9°, θ_min_ = 1.9°
ω scans	*h* = −11→9
Absorption correction: analytical (*XPREP*; Bruker, 2007)	*k* = −16→15
*T*_min_ = 0.149, *T*_max_ = 0.281	*l* = −14→20
9353 measured reflections	

### Refinement

**Table:**

Refinement on *F*^2^	Primary atom site location: structure-invariant direct methods
Least-squares matrix: full	Secondary atom site location: difference Fourier map
*R*[*F*^2^ > 2σ(*F*^2^)] = 0.027	Hydrogen site location: inferred from neighbouring sites
*wR*(*F*^2^) = 0.056	H-atom parameters constrained
*S* = 0.99	*w* = 1/[σ^2^(*F*_o_^2^) + (0.0257*P*)^2^] where *P* = (*F*_o_^2^ + 2*F*_c_^2^)/3
6948 reflections	(Δ/σ)_max_ = 0.003
293 parameters	Δρ_max_ = 0.88 e Å^−^^3^
0 restraints	Δρ_min_ = −0.64 e Å^−^^3^

### Special details

**Table:**

Geometry. All e.s.d.'s (except the e.s.d. in the dihedral angle between two l.s. planes) are estimated using the full covariance matrix. The cell e.s.d.'s are taken into account individually in the estimation of e.s.d.'s in distances, angles and torsion angles; correlations between e.s.d.'s in cell parameters are only used when they are defined by crystal symmetry. An approximate (isotropic) treatment of cell e.s.d.'s is used for estimating e.s.d.'s involving l.s. planes.
Refinement. Refinement of *F*^2^ against ALL reflections. The weighted *R*-factor *wR* and goodness of fit *S* are based on *F*^2^, conventional *R*-factors *R* are based on *F*, with *F* set to zero for negative *F*^2^. The threshold expression of *F*^2^ > 2σ(*F*^2^) is used only for calculating *R*-factors(gt) *etc*. and is not relevant to the choice of reflections for refinement. *R*-factors based on *F*^2^ are statistically about twice as large as those based on *F*, and *R*- factors based on ALL data will be even larger.

### Fractional atomic coordinates and isotropic or equivalent isotropic displacement parameters (Å^2^)

**Table:**

	*x*	*y*	*z*	*U*_iso_*/*U*_eq_	
U1	0.488854 (15)	0.690915 (11)	0.372080 (8)	0.03519 (4)	
O1	0.3391 (3)	0.5504 (2)	0.3581 (2)	0.0535 (6)	
O2	0.6386 (3)	0.8327 (2)	0.38786 (19)	0.0525 (6)	
O3	0.6977 (3)	0.6473 (3)	0.47398 (18)	0.0566 (7)	
O4	0.6471 (3)	0.5741 (2)	0.27211 (18)	0.0521 (6)	
O5	0.3569 (4)	0.6784 (3)	0.21310 (17)	0.0579 (7)	
O6	0.2846 (3)	0.8105 (3)	0.38825 (18)	0.0567 (7)	
N1	0.4613 (4)	0.7730 (3)	0.55740 (19)	0.0397 (6)	
C1	0.9705 (6)	0.6794 (5)	0.5706 (3)	0.0810 (14)	
H1A	0.9878	0.7680	0.6071	0.121*	
H1B	1.0774	0.6618	0.5601	0.121*	
H1C	0.9231	0.6316	0.6080	0.121*	
C2	0.8488 (5)	0.6426 (3)	0.4705 (3)	0.0493 (9)	
C3	0.9047 (5)	0.6043 (4)	0.3827 (3)	0.0573 (10)	
H3	1.0188	0.6043	0.3872	0.069*	
C4	0.8004 (5)	0.5661 (3)	0.2886 (3)	0.0497 (9)	
C5	0.8646 (7)	0.5049 (5)	0.1981 (4)	0.0757 (14)	
H5A	0.7867	0.4249	0.1584	0.114*	
H5B	0.9744	0.4920	0.2186	0.114*	
H5C	0.8738	0.5586	0.1595	0.114*	
C6	0.2066 (7)	0.6847 (5)	0.0645 (3)	0.0796 (15)	
H6A	0.3049	0.7172	0.0433	0.119*	
H6B	0.1140	0.7170	0.0434	0.119*	
H6C	0.1752	0.5940	0.0354	0.119*	
C7	0.2466 (5)	0.7251 (4)	0.1764 (3)	0.0496 (9)	
C8	0.1637 (5)	0.8067 (4)	0.2321 (3)	0.0560 (10)	
H8	0.0878	0.8375	0.1980	0.067*	
C9	0.1856 (4)	0.8459 (3)	0.3350 (3)	0.0473 (8)	
C10	0.0891 (6)	0.9354 (4)	0.3879 (3)	0.0670 (12)	
H10A	−0.0194	0.8881	0.3894	0.101*	
H10B	0.0723	0.9919	0.3531	0.101*	
H10C	0.1526	0.9833	0.4549	0.101*	
C11	0.4545 (5)	0.6962 (3)	0.6101 (3)	0.0449 (8)	
H11	0.4583	0.6129	0.5791	0.054*	
C12	0.4422 (5)	0.7361 (4)	0.7083 (3)	0.0479 (8)	
H12	0.4371	0.6798	0.7421	0.058*	
C13	0.4373 (4)	0.8594 (3)	0.7568 (2)	0.0434 (8)	
C14	0.4484 (5)	0.9381 (3)	0.7028 (3)	0.0476 (8)	
H14	0.4501	1.0227	0.7333	0.057*	
C15	0.4570 (5)	0.8929 (3)	0.6042 (3)	0.0470 (8)	
H15	0.4599	0.9474	0.5688	0.056*	
C16	0.4135 (6)	0.9028 (4)	0.8628 (3)	0.0576 (10)	
H16A	0.4701	0.9919	0.8956	0.069*	
H16B	0.4675	0.8568	0.8984	0.069*	
C17	0.2268 (6)	0.8831 (4)	0.8692 (3)	0.0586 (10)	
H17A	0.1655	0.7980	0.8253	0.070*	
H17B	0.2195	0.8901	0.9367	0.070*	
C18	0.1404 (5)	0.9765 (4)	0.8412 (3)	0.0536 (9)	
H18A	0.1565	0.9747	0.7759	0.064*	
H18B	0.0192	0.9492	0.8361	0.064*	
C19	0.2056 (5)	1.1116 (4)	0.9144 (3)	0.0488 (8)	
C20	0.1846 (6)	1.1420 (4)	1.0114 (3)	0.0621 (11)	
H20	0.1317	1.0788	1.0318	0.075*	
C21	0.2424 (6)	1.2672 (5)	1.0796 (3)	0.0750 (14)	
H21	0.2255	1.2868	1.1442	0.090*	
C22	0.3239 (6)	1.3604 (5)	1.0503 (4)	0.0772 (14)	
H22	0.3654	1.4431	1.0952	0.093*	
C23	0.3428 (7)	1.3292 (5)	0.9534 (4)	0.0796 (14)	
H23	0.3952	1.3923	0.9328	0.096*	
C24	0.2867 (6)	1.2082 (4)	0.8869 (3)	0.0627 (11)	
H24	0.3030	1.1900	0.8222	0.075*	

### Atomic displacement parameters (Å^2^)

**Table:**

	*U*^11^	*U*^22^	*U*^33^	*U*^12^	*U*^13^	*U*^23^
U1	0.03593 (7)	0.03608 (7)	0.03601 (7)	0.01545 (5)	0.00830 (5)	0.01242 (5)
O1	0.0494 (15)	0.0444 (15)	0.0616 (16)	0.0058 (12)	0.0129 (12)	0.0154 (12)
O2	0.0563 (16)	0.0411 (14)	0.0611 (16)	0.0096 (12)	0.0197 (13)	0.0182 (12)
O3	0.0536 (16)	0.078 (2)	0.0503 (14)	0.0391 (15)	0.0132 (12)	0.0262 (14)
O4	0.0537 (16)	0.0575 (16)	0.0478 (14)	0.0285 (13)	0.0151 (12)	0.0124 (12)
O5	0.0669 (18)	0.0722 (19)	0.0417 (13)	0.0421 (15)	0.0081 (12)	0.0175 (13)
O6	0.0607 (17)	0.0762 (19)	0.0453 (13)	0.0456 (15)	0.0134 (12)	0.0208 (13)
N1	0.0469 (16)	0.0383 (15)	0.0385 (14)	0.0166 (13)	0.0148 (12)	0.0141 (12)
C1	0.067 (3)	0.093 (4)	0.075 (3)	0.026 (3)	−0.012 (2)	0.029 (3)
C2	0.046 (2)	0.043 (2)	0.062 (2)	0.0183 (16)	0.0049 (17)	0.0212 (17)
C3	0.0358 (19)	0.065 (3)	0.079 (3)	0.0201 (18)	0.0187 (19)	0.030 (2)
C4	0.052 (2)	0.044 (2)	0.068 (2)	0.0230 (17)	0.0303 (19)	0.0257 (18)
C5	0.093 (4)	0.072 (3)	0.086 (3)	0.043 (3)	0.054 (3)	0.032 (3)
C6	0.092 (4)	0.107 (4)	0.046 (2)	0.048 (3)	0.004 (2)	0.029 (2)
C7	0.048 (2)	0.056 (2)	0.0462 (19)	0.0167 (18)	0.0018 (16)	0.0232 (17)
C8	0.055 (2)	0.064 (3)	0.052 (2)	0.030 (2)	0.0006 (18)	0.0204 (19)
C9	0.0377 (18)	0.043 (2)	0.058 (2)	0.0173 (15)	0.0035 (16)	0.0130 (17)
C10	0.062 (3)	0.068 (3)	0.071 (3)	0.040 (2)	0.011 (2)	0.014 (2)
C11	0.057 (2)	0.0391 (19)	0.0473 (19)	0.0229 (16)	0.0172 (17)	0.0184 (15)
C12	0.060 (2)	0.049 (2)	0.0470 (19)	0.0214 (18)	0.0182 (17)	0.0262 (17)
C13	0.0427 (19)	0.048 (2)	0.0362 (16)	0.0154 (16)	0.0057 (14)	0.0101 (15)
C14	0.060 (2)	0.0379 (19)	0.0438 (18)	0.0158 (17)	0.0158 (17)	0.0096 (15)
C15	0.062 (2)	0.0379 (19)	0.0488 (19)	0.0157 (17)	0.0215 (17)	0.0192 (16)
C16	0.076 (3)	0.066 (3)	0.0361 (18)	0.034 (2)	0.0081 (18)	0.0178 (18)
C17	0.079 (3)	0.055 (2)	0.051 (2)	0.021 (2)	0.029 (2)	0.0227 (19)
C18	0.052 (2)	0.057 (2)	0.053 (2)	0.0113 (18)	0.0174 (18)	0.0191 (19)
C19	0.0391 (19)	0.058 (2)	0.048 (2)	0.0161 (17)	0.0076 (16)	0.0160 (18)
C20	0.063 (3)	0.065 (3)	0.056 (2)	0.015 (2)	0.018 (2)	0.017 (2)
C21	0.073 (3)	0.084 (4)	0.058 (3)	0.033 (3)	0.011 (2)	0.007 (2)
C22	0.069 (3)	0.051 (3)	0.094 (4)	0.018 (2)	−0.001 (3)	0.009 (3)
C23	0.081 (3)	0.053 (3)	0.103 (4)	0.013 (2)	0.019 (3)	0.029 (3)
C24	0.064 (3)	0.063 (3)	0.069 (3)	0.021 (2)	0.019 (2)	0.030 (2)

### Geometric parameters (Å, º)

**Table:**

U1—O1	1.773 (3)	C10—H10A	0.9600
U1—O2	1.777 (3)	C10—H10B	0.9600
U1—O3	2.330 (2)	C10—H10C	0.9600
U1—O4	2.360 (2)	C11—C12	1.376 (5)
U1—O5	2.348 (2)	C11—H11	0.9300
U1—O6	2.354 (2)	C12—C13	1.382 (5)
U1—N1	2.610 (3)	C12—H12	0.9300
O3—C2	1.260 (4)	C13—C14	1.375 (5)
O4—C4	1.272 (4)	C13—C16	1.512 (5)
O5—C7	1.271 (4)	C14—C15	1.375 (5)
O6—C9	1.251 (4)	C14—H14	0.9300
N1—C11	1.342 (4)	C15—H15	0.9300
N1—C15	1.342 (4)	C16—C17	1.528 (6)
C1—C2	1.519 (5)	C16—H16A	0.9700
C1—H1A	0.9600	C16—H16B	0.9700
C1—H1B	0.9600	C17—C18	1.519 (5)
C1—H1C	0.9600	C17—H17A	0.9700
C2—C3	1.384 (6)	C17—H17B	0.9700
C3—C4	1.386 (6)	C18—C19	1.517 (6)
C3—H3	0.9300	C18—H18A	0.9700
C4—C5	1.501 (5)	C18—H18B	0.9700
C5—H5A	0.9600	C19—C20	1.382 (5)
C5—H5B	0.9600	C19—C24	1.389 (6)
C5—H5C	0.9600	C20—C21	1.407 (6)
C6—C7	1.505 (5)	C20—H20	0.9300
C6—H6A	0.9600	C21—C22	1.375 (7)
C6—H6B	0.9600	C21—H21	0.9300
C6—H6C	0.9600	C22—C23	1.375 (7)
C7—C8	1.385 (5)	C22—H22	0.9300
C8—C9	1.388 (5)	C23—C24	1.362 (6)
C8—H8	0.9300	C23—H23	0.9300
C9—C10	1.504 (5)	C24—H24	0.9300
			
O1—U1—O2	179.19 (11)	O6—C9—C8	122.9 (3)
O1—U1—O3	91.86 (12)	O6—C9—C10	116.8 (3)
O1—U1—O4	91.38 (11)	C8—C9—C10	120.3 (3)
O1—U1—O5	89.82 (12)	C9—C10—H10A	109.5
O1—U1—O6	92.85 (12)	C9—C10—H10B	109.5
O2—U1—O3	87.91 (12)	H10A—C10—H10B	109.5
O2—U1—O4	89.27 (11)	C9—C10—H10C	109.5
O2—U1—O5	90.77 (12)	H10A—C10—H10C	109.5
O2—U1—O6	86.82 (11)	H10B—C10—H10C	109.5
O3—U1—O4	70.88 (9)	N1—C11—C12	122.5 (3)
O3—U1—O5	149.99 (9)	N1—C11—H11	118.8
O3—U1—O6	138.83 (9)	C12—C11—H11	118.8
O4—U1—O5	79.13 (9)	C11—C12—C13	120.3 (3)
O4—U1—O6	149.71 (9)	C11—C12—H12	119.8
O5—U1—O6	70.91 (9)	C13—C12—H12	119.8
O1—U1—N1	86.45 (11)	C14—C13—C12	116.7 (3)
O2—U1—N1	92.74 (11)	C14—C13—C16	122.3 (3)
O3—U1—N1	69.37 (9)	C12—C13—C16	121.0 (3)
O4—U1—N1	140.08 (8)	C15—C14—C13	120.7 (3)
O5—U1—N1	140.62 (9)	C15—C14—H14	119.6
O6—U1—N1	70.15 (9)	C13—C14—H14	119.6
C2—O3—U1	132.2 (2)	N1—C15—C14	122.3 (3)
C4—O4—U1	132.7 (2)	N1—C15—H15	118.9
C7—O5—U1	137.4 (2)	C14—C15—H15	118.9
C9—O6—U1	139.2 (2)	C13—C16—C17	113.2 (3)
C11—N1—C15	117.5 (3)	C13—C16—H16A	108.9
C11—N1—U1	120.6 (2)	C17—C16—H16A	108.9
C15—N1—U1	121.9 (2)	C13—C16—H16B	108.9
C2—C1—H1A	109.5	C17—C16—H16B	108.9
C2—C1—H1B	109.5	H16A—C16—H16B	107.7
H1A—C1—H1B	109.5	C18—C17—C16	113.9 (3)
C2—C1—H1C	109.5	C18—C17—H17A	108.8
H1A—C1—H1C	109.5	C16—C17—H17A	108.8
H1B—C1—H1C	109.5	C18—C17—H17B	108.8
O3—C2—C3	123.9 (4)	C16—C17—H17B	108.8
O3—C2—C1	115.6 (4)	H17A—C17—H17B	107.7
C3—C2—C1	120.5 (4)	C19—C18—C17	114.2 (3)
C2—C3—C4	123.8 (3)	C19—C18—H18A	108.7
C2—C3—H3	118.1	C17—C18—H18A	108.7
C4—C3—H3	118.1	C19—C18—H18B	108.7
O4—C4—C3	124.5 (3)	C17—C18—H18B	108.7
O4—C4—C5	115.8 (4)	H18A—C18—H18B	107.6
C3—C4—C5	119.7 (4)	C20—C19—C24	117.9 (4)
C4—C5—H5A	109.5	C20—C19—C18	120.4 (4)
C4—C5—H5B	109.5	C24—C19—C18	121.7 (4)
H5A—C5—H5B	109.5	C19—C20—C21	120.9 (4)
C4—C5—H5C	109.5	C19—C20—H20	119.5
H5A—C5—H5C	109.5	C21—C20—H20	119.5
H5B—C5—H5C	109.5	C22—C21—C20	119.7 (5)
C7—C6—H6A	109.5	C22—C21—H21	120.1
C7—C6—H6B	109.5	C20—C21—H21	120.1
H6A—C6—H6B	109.5	C21—C22—C23	118.8 (5)
C7—C6—H6C	109.5	C21—C22—H22	120.6
H6A—C6—H6C	109.5	C23—C22—H22	120.6
H6B—C6—H6C	109.5	C24—C23—C22	121.7 (5)
O5—C7—C8	124.5 (3)	C24—C23—H23	119.1
O5—C7—C6	115.5 (4)	C22—C23—H23	119.1
C8—C7—C6	120.0 (3)	C23—C24—C19	120.8 (4)
C7—C8—C9	124.8 (3)	C23—C24—H24	119.6
C7—C8—H8	117.6	C19—C24—H24	119.6
C9—C8—H8	117.6		
